# The Rice CBP60 Protein OsCBP609 Interacts With Pik1‐H4 and Supports Salicylic Acid‐Responsive Immunity Against *Magnaporthe oryzae*

**DOI:** 10.1111/mpp.70384

**Published:** 2026-11-08

**Authors:** Huabin Xie, Danhong Wei, Jiayang Li, Chunzi Lin, Xiaodi Zou, Renhui Li, Yan Hou, Xianya Jiang, Chun Chen, Tao Guo, Jiafeng Wang

**Affiliations:** ^1^ National Engineering Research Center of Plant Space Breeding, Basic Research Center of Excellence for Precise Breeding of Future Crops, Guangdong Key Laboratory of Plant Molecular Breeding South China Agricultural University Guangzhou Guangdong China; ^2^ Yangjiang Institute of Agricultural Sciences Yangjiang Guangdong China

**Keywords:** OsCBP609, Pik1‐H4, rice blast, salicylic acid signalling

## Abstract

Plant immunity depends on coordination between immune receptors and hormone‐responsive transcriptional networks, yet how CBP60 proteins contribute to disease resistance in monocots remains poorly understood. Here, we characterized OsCBP609, a rice CBP60 family protein involved in resistance to *Magnaporthe oryzae*. Expression of *OsCBP609* was responsive to *M. oryzae* infection and defence‐related signals, and the encoded protein localized to the nucleus and exhibited transcriptional activation. CRISPR/Cas9‐mediated disruption of *OsCBP609* increased lesion development and fungal biomass following infection with multiple *M. oryzae* isolates. Transcriptome profiling showed that loss of *OsCBP609* was associated with altered expression of genes involved in plant–pathogen interactions and hormone‐related signalling pathways during infection. Although basal salicylic acid (SA) levels were elevated in the *oscbp609* mutant, SA‐responsive defence transcription was attenuated, and exogenous SA failed to restore resistance to the wild‐type level, supporting a role for OsCBP609 in the transcriptional response to SA. OsCBP609 physically interacted with the NLR protein Pik1‐H4 in the nucleus, and co‐expression of the two proteins produced significantly higher reporter activity than expression of either protein alone. Disruption of *OsCBP609* in a *Pik‐H4* near‐isogenic line also reduced blast resistance, supporting a functional contribution of OsCBP609 to Pik‐H4‐associated immunity. Together, these results identify OsCBP609 as a CBP60 transcriptional regulator that supports SA‐responsive defence and Pik‐H4‐associated resistance to rice blast.

## Graphical abstract

OsCBP609 is required for effective rice blast resistance. It supports salicylic acid‐responsive defence transcription, interacts with the Pik1‐H4 NLR protein and contributes to Pik‐H4‐mediated immunity, revealing a functional link between CBP60‐dependent defence regulation and NLR‐associated resistance in rice.

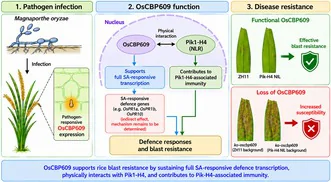

## 1 Introduction

Rice ( *Oryza sativa* ) is a major staple crop that supports nearly half of the global population (Elert 2014). However, rice production is severely threatened by blast disease, caused by the filamentous fungus *Magnaporthe oryzae*, which can result in substantial yield losses worldwide (Azizi et al. 2016). Plants defend themselves against pathogen infection through interconnected immune pathways initiated by cell‐surface pattern‐recognition receptors and intracellular nucleotide‐binding leucine‐rich repeat receptors (NLRs). These pathways activate a range of downstream responses, including Ca^2+^ influx, mitogen‐activated protein kinase cascades, transcriptional reprogramming and phytohormone‐mediated defence signalling (Jones and Dangl 2006; Dodds and Rathjen 2010; Yuan, Jiang, et al. 2021; Yuan, Ngou, et al. 2021; Ngou et al. 2021). Understanding how these signalling components are coordinated is therefore important for elucidating the molecular basis of rice immunity to *M. oryzae* .

Ca^2+^ functions as a central second messenger in plant immune signalling (Du et al. 2009; Boudsocq et al. 2010). Changes in cytosolic Ca^2+^ concentrations are perceived primarily by calmodulins (CaMs) and CaM‐like proteins (CMLs), which transmit the signal through interactions with downstream calmodulin‐binding proteins (CaMBPs) (DeFalco et al. 2010; Zeng et al. 2015). The calmodulin‐binding protein 60 (CBP60) family comprises plant‐specific transcriptional regulators with important functions in immunity. In *Arabidopsis thaliana* , CBP60g and its close homologue SARD1 directly activate genes involved in salicylic acid (SA) biosynthesis and defence, thereby promoting pathogen‐induced SA accumulation and disease resistance (Zhang et al. 2010; Sun et al. 2015). By contrast, CBP60a negatively regulates immunity, illustrating the functional diversification of this protein family (Truman et al. 2013). Genetic studies have further shown that simultaneous loss of CBP60g and SARD1 strongly compromises pathogen‐induced SA accumulation and immune responses (Wang et al. 2011; Sun et al. 2015).

CBP60 homologues have also been implicated in immune regulation in several crop species. CBP60‐like proteins participate in defence responses in soybean and cotton, whereas CBP60b orthologs from tomato and cucumber can activate defence‐associated genes and functionally complement the corresponding *Arabidopsis* mutant (Hu et al. 2020; Ye et al. 2024; Li et al. 2024). In addition, *Arabidopsis* CBP60b has been linked to NLR‐mediated immune surveillance, suggesting a potential connection between CBP60 transcriptional regulators and intracellular immune receptors (Li et al. 2021). Nevertheless, the functions of CBP60 proteins in monocots remain poorly understood. Rice maintains relatively high basal SA levels and possesses distinctive SA‐associated defence regulation (Silverman et al. 1995; Xu et al. 2023), raising the question of whether rice CBP60 proteins primarily influence SA accumulation, downstream SA responsiveness, or other immune pathways. It also remains unclear whether CBP60 proteins functionally interact with NLR‐mediated resistance in rice. The *Pik* locus encodes paired NLRs, and AVR‐Pik recognition is mediated by direct interaction with the integrated HMA domain of Pik‐1 proteins (Ashikawa et al. 2008; Kanzaki et al. 2012; Maqbool et al. 2015). *Pik‐H4* is a paired NLR locus, and Pik1‐H4, one of its encoded NLR proteins, provides a suitable system for investigating the possible association between CBP60 proteins and NLR‐dependent immunity (Xiao et al. 2011; Gu et al. 2025).

Here, we characterized *OsCBP609*, a rice CBP60 family gene encoding a nuclear‐localized transcriptional activator. Disruption of *OsCBP609* increased susceptibility to multiple *M. oryzae* isolates without causing detectable changes in major agronomic traits. Although endogenous SA accumulated to higher levels in the *oscbp609* mutant, pathogen‐induced defence gene expression and SA‐induced PR gene expression were attenuated, indicating that OsCBP609 is required for effective SA‐responsive defence rather than SA accumulation itself. OsCBP609 also interacted with the NLR protein Pik1‐H4 in the nucleus, and co‐expression of the two proteins produced significantly higher reporter activity than expression of either protein alone. Moreover, disruption of *OsCBP609* in a Pik‐H4 near‐isogenic line reduced blast resistance. Together, these findings identify OsCBP609 as a CBP60 transcriptional regulator that supports SA‐responsive immunity and contributes to Pik‐H4‐associated resistance against rice blast.

## 2 Results

### 2.1 Expression Patterns of *OsCBP609*

In our previous transcriptome analysis comparing resistant and susceptible rice cultivars in response to *M. oryzae* infection, we identified a set of differentially expressed genes (DEGs) associated with blast resistance. Among them, a calmodulin‐binding protein family gene, *OsCBP609*, was markedly responsive to pathogen challenge and thus selected for further functional characterization. *OsCBP609* is located on chromosome 2 and encodes a protein containing a conserved calmodulin‐binding domain (Figure 1a). Phylogenetic analysis placed OsCBP609 within a well‐supported clade associated with *Arabidopsis* CBP60b, CBP60c and CBP60d (Figure 1b).

**FIGURE 1:**
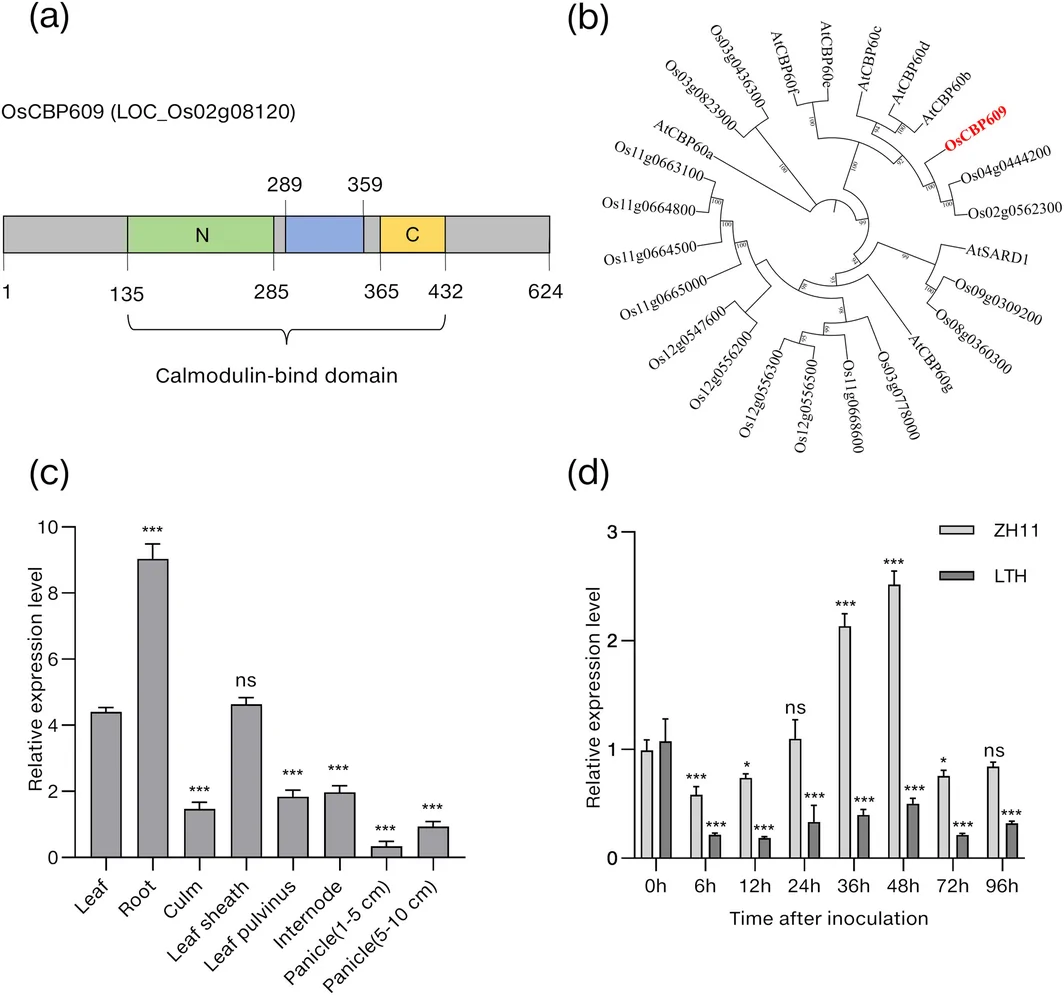
Phylogenetic and expression analysis of *OsCBP609*. (a) Protein domain architecture of OsCBP609 analysed using the UniProt database. The calmodulin‐binding domain comprises the N‐terminal, central and C‐terminal subdomains, indicated by green, blue and yellow shading, respectively. (b) Maximum‐likelihood phylogenetic tree of CBP60‐related proteins from rice and *Arabidopsis thaliana* . Bootstrap values ≥ 70 are shown at the internal nodes. OsCBP609 is highlighted in red. (c) Tissue‐specific expression patterns of *OsCBP609* determined by reverse transcription‐quantitative PCR (RT‐qPCR) in leaf, root, culm, leaf sheath, leaf pulvinus, internode, panicle (1–5 cm) and panicle (5–10 cm). (d) Time‐course expression profiles of *OsCBP609* in Zhonghua 11 (ZH11) and Lijiangxintuanheigu (LTH) following inoculation with *Magnaporthe oryzae* strain GDYJ7, analysed by RT‐qPCR. For (c) and (d), *Actin* was used as an internal reference. Data represent mean ± SD of three biological replicates. Significant differences are indicated as follows: * *p* < 0.05, ***p < 0.001; ns, not significant (Student's *t*‐test).

Reverse transcription‐quantitative PCR (RT‐qPCR) analysis revealed that *OsCBP609* was expressed in different rice tissues, with relatively high expression in roots and leaves (Figure 1c). Upon inoculation with *M. oryzae* isolate GDYJ7, *OsCBP609* expression in the susceptible cultivar Lijiangxintuanheigu (LTH) was initially suppressed but gradually increased, peaking at 48 h post‐inoculation (hpi). In contrast, expression in the resistant cultivar Zhonghua 11 (ZH11) was transiently repressed at early stages, recovered to basal levels at 24 hpi, reached a maximum at 48 hpi and then declined (Figure 1d). These results indicate that *OsCBP609* is transcriptionally responsive to *M. oryzae* infection.

### 2.2 OsCBP609 Localizes to the Nucleus and Functions as a Transcriptional Activator

To investigate the subcellular localization of OsCBP609, we constructed a pAN580‐OsCBP609‐GFP fusion vector and co‐transformed it into rice protoplasts together with a nuclear marker (RFP). The OsCBP609‐GFP signal overlapped with the nuclear RFP fluorescence (Figure 2a), indicating that OsCBP609 is a nuclear protein. To assess its transcriptional activity, we expressed pGBKT7‐Empty, pGBKT7‐IPA1 (positive control) (Wang et al. 2025) and pGBKT7‐OsCBP609 in the yeast strain Y2H Gold. Both pGBKT7‐IPA1 and pGBKT7‐OsCBP609 supported yeast growth on SD/−Trp/−His and SD/−Trp/−His/−Ade media and developed blue coloration on SD/−Trp/−His/−Ade medium supplemented with X‐α‐Gal, whereas the empty‐vector control did not (Figure 2b). These results confirm that OsCBP609 possesses transcriptional activation activity. This finding was further validated using a *Nicotiana benthamiana* luciferase (LUC) reporter assay, where the pBD‐OsCBP609 effector produced significantly higher relative LUC intensity than the pBD‐Empty control (Figure 2c,d). Together, these results demonstrate that OsCBP609 localizes to the nucleus and acts as a transcriptional activator.

**FIGURE 2:**
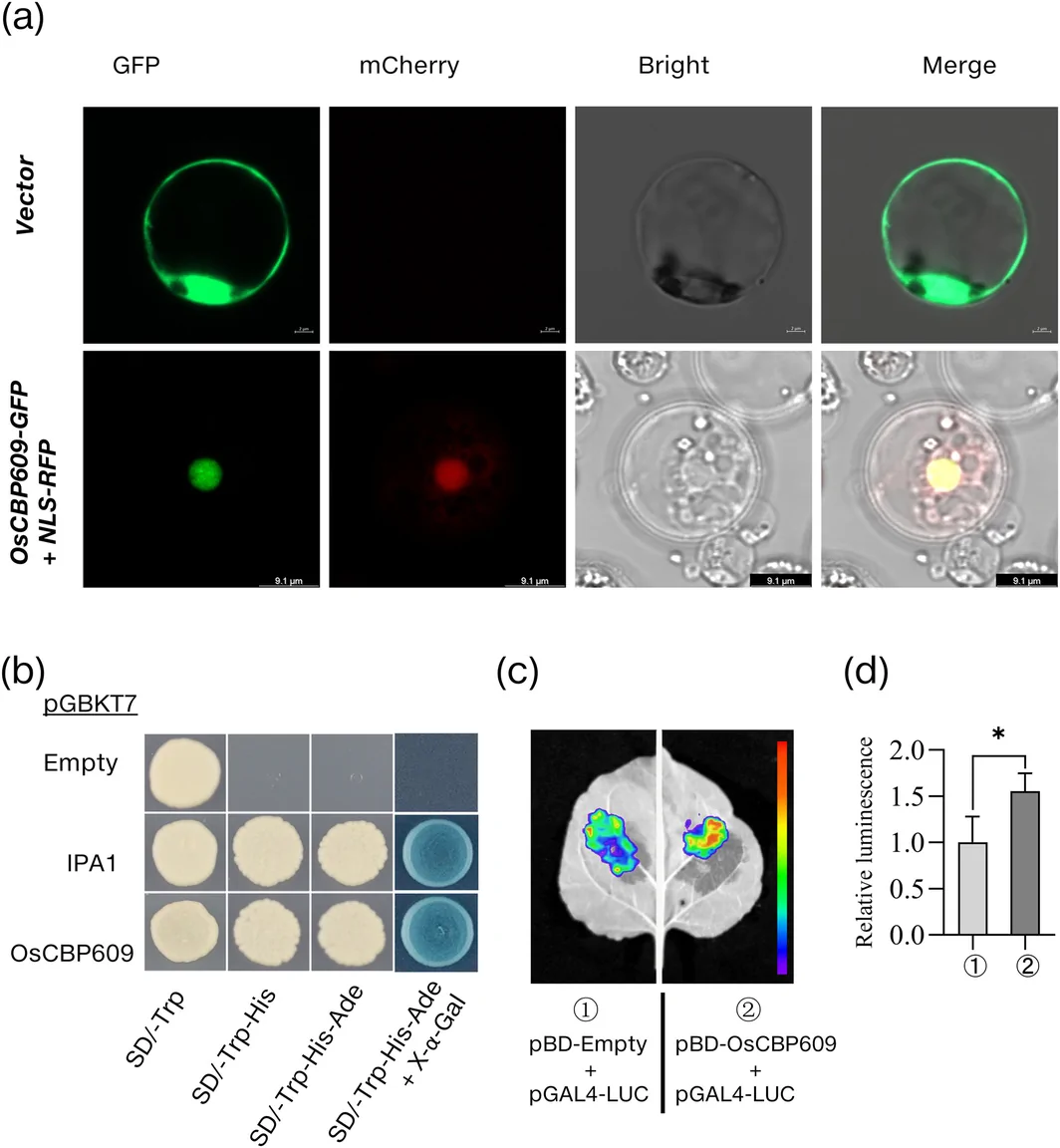
Subcellular localization and transcriptional activity of OsCBP609. (a) Subcellular localization of OsCBP609 in rice protoplasts examined by confocal microscopy. Free GFP was present throughout the cell, while OsCBP609‐GFP was exclusively localized in the nucleus. (b) Transcriptional activation assay of OsCBP609 in yeast. Y2HGold cells carrying pGBKT7‐Empty, pGBKT7‐IPA1 (positive control), or pGBKT7‐OsCBP609 were grown on SD/−Trp, SD/−Trp/−His, SD/−Trp/−His/−Ade and SD/−Trp/−His/−Ade + X‐α‐Gal. Blue coloration indicates activation of the X‐α‐Gal‐responsive reporter. (c) Transcriptional activation activity of OsCBP609 assessed by transient luciferase imaging assay in *Nicotiana benthamiana* leaves. (d) Quantification of the luminescence signals shown in (c). Data are presented as mean ± SD (*n* = 3). *p < 0.05.

### 2.3 *OsCBP609* Is Required for Resistance to *M. oryzae*

To examine the biological function of *OsCBP609*, we generated two *OsCBP609* knockout (*ko‐oscbp609‐1*, *ko‐oscbp609‐2*) and two *OsCBP609‐*overexpressing lines (OE‐OsCBP609‐1, OE‐OsCBP609‐2). Sequence analysis confirmed the CRISPR/Cas9‐induced mutations in the two *oscbp609* lines (Figure 3a), whereas RT‐qPCR confirmed elevated *OsCBP609* expression in the overexpression lines (Figure 3b). Detached‐leaf inoculation assays revealed that blast isolates Zhong1, Guy11 and ZJ21X18 produced significantly larger lesions on the knockout lines than on the wild type (WT), whereas lesion development in the overexpression lines was comparable to the WT (Figure 3c–i). Consistently, fungal biomass was markedly increased in the knockout plants but remained unchanged in the overexpression lines (Figure 3k–m). Moreover, the expression of the defence‐related marker genes *OsPR1a*, *OsPR1b* and *OsPAL1* was significantly reduced in the knockout lines at 24 hpi after ZJ21X18 spray inoculation (Figure S1). At 7 days post‐inoculation (dpi), knockout plants exhibited a greater number of lesions, which were also larger in size compared with those on the WT plants (Figure 3j). Together, these results indicate that *OsCBP609* is required for effective resistance to *M. oryzae* . No significant differences in the examined agronomic traits, including plant height, grain length, grain width and 1000‐grain weight, were detected between the transgenic and WT plants (Figure S2).

**FIGURE 3:**
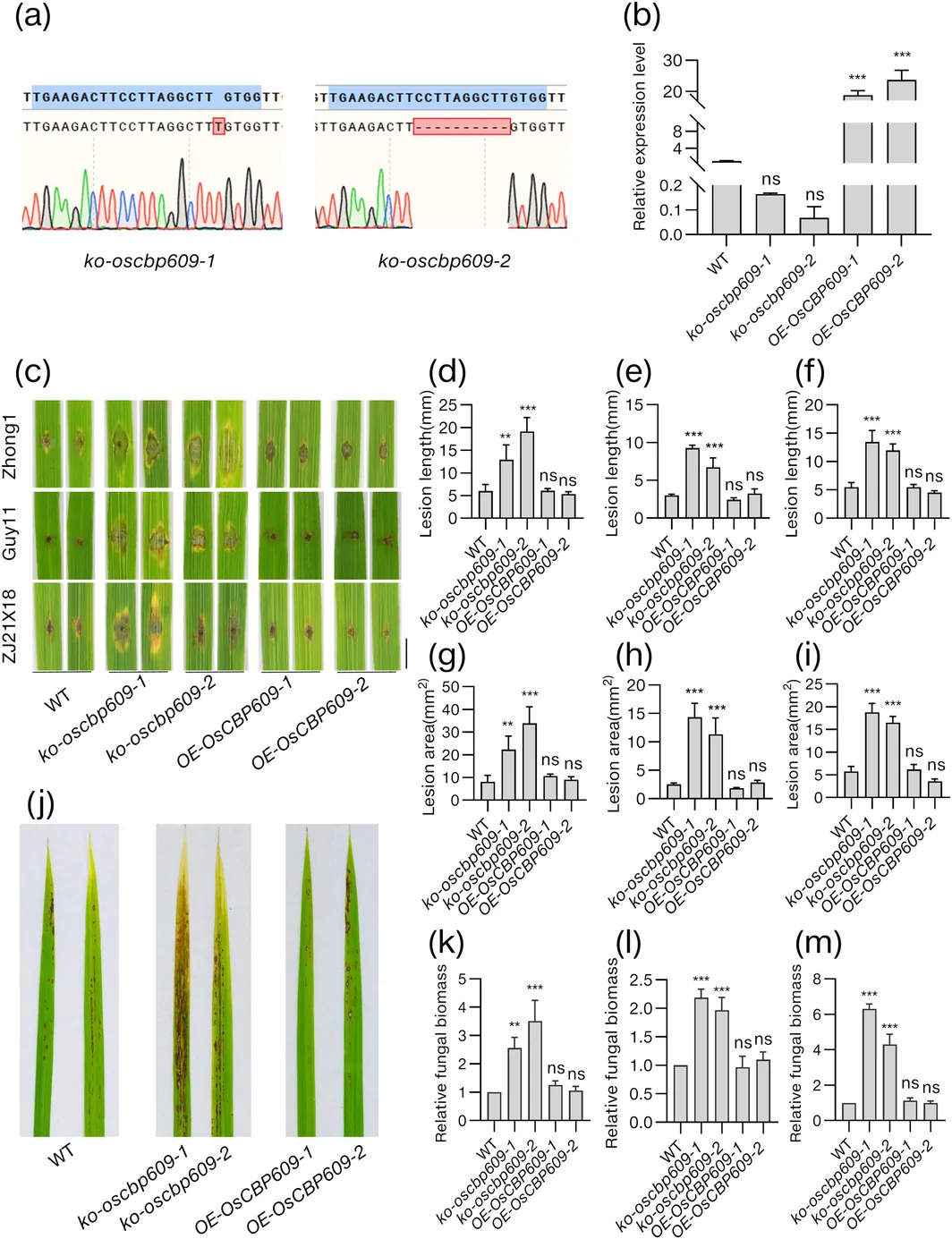
Loss of *OsCBP609* increases susceptibility to rice blast. (a) Genotyping of CRISPR/Cas9‐generated *oscbp609* knockout mutants. Target sequences are highlighted in blue; *ko‐oscbp609‐1* carries a T insertion, and *ko‐oscbp609‐2* carries a 10‐bp deletion. (b) Expression levels of *OsCBP609* in wild type (WT), mutant and overexpression (OE) lines analysed by reverse transcription‐quantitative PCR. (c) Lesions on detached rice leaves inoculated with *Magnaporthe oryzae* isolates Zhong1, Guy11 and ZJ21X18 at 7 days post‐inoculation. Scale bar = 1 cm. (d–f) Lesion lengths on detached leaves inoculated with isolates Zhong1, Guy11 and ZJ21X18, respectively. (g–i) Lesion areas on detached leaves inoculated with isolates Zhong1, Guy11 and ZJ21X18, respectively. (j) Disease phenotypes of WT, *oscbp609* knockout mutants and overexpression lines after spray inoculation with ZJ21X18. (k–m) Relative fungal biomass on detached leaves inoculated with isolates (k) Zhong1, (l) Guy11 and (m) ZJ21X18. Data are presented as mean ± SD (*n* = 3). Significant differences are indicated as follows: **p < 0.01, ***p < 0.001; ns, not significant (Student's *t*‐test).

### 2.4 Loss of *OsCBP609* Is Associated With Altered Defence‐Related Transcription During Infection

To further elucidate the regulatory role of *OsCBP609*, we performed transcriptome profiling of *OsCBP609* knockout and WT (ZH11) plants upon *M. oryzae* infection. A total of 2275 DEGs were identified (Table S1). To assess the reliability of the RNA‐seq dataset, eight DEGs were randomly selected for RT‐qPCR validation; their expression trends were consistent with the RNA‐seq results (Figure S3).

In the ko‐0h_VS_ZH11‐0 h comparison, 957 DEGs were identified (Figure 4a), of which 750 were not detected in either the ko‐12h_VS_ZH11‐12 h or ko‐24h_VS_ZH11‐24 h comparison. These 750 genes were therefore considered basal genotype‐dependent DEGs rather than infection‐responsive DEGs and were analysed separately (Figure S4). The remaining 1525 DEGs were used for the GO functional classification and KEGG enrichment analyses shown in Figure 4b,c. GO functional classification showed that these genes were mainly assigned to biological processes including regulation of DNA‐templated transcription and response to stimulus, cellular components such as the nucleus and extracellular region, and molecular functions including DNA‐binding transcription factor activity and sequence‐specific DNA binding (Figure 4c). KEGG pathway analysis highlighted plant hormone signal transduction (ko04075), MAPK signalling pathway–plant (ko04016) and plant–pathogen interaction (ko04626) as the most significantly enriched pathways (Figure 4b). By contrast, plant hormone signal transduction was not significantly enriched among the 750 basal genotype‐dependent DEGs (Figure S4).

**FIGURE 4:**
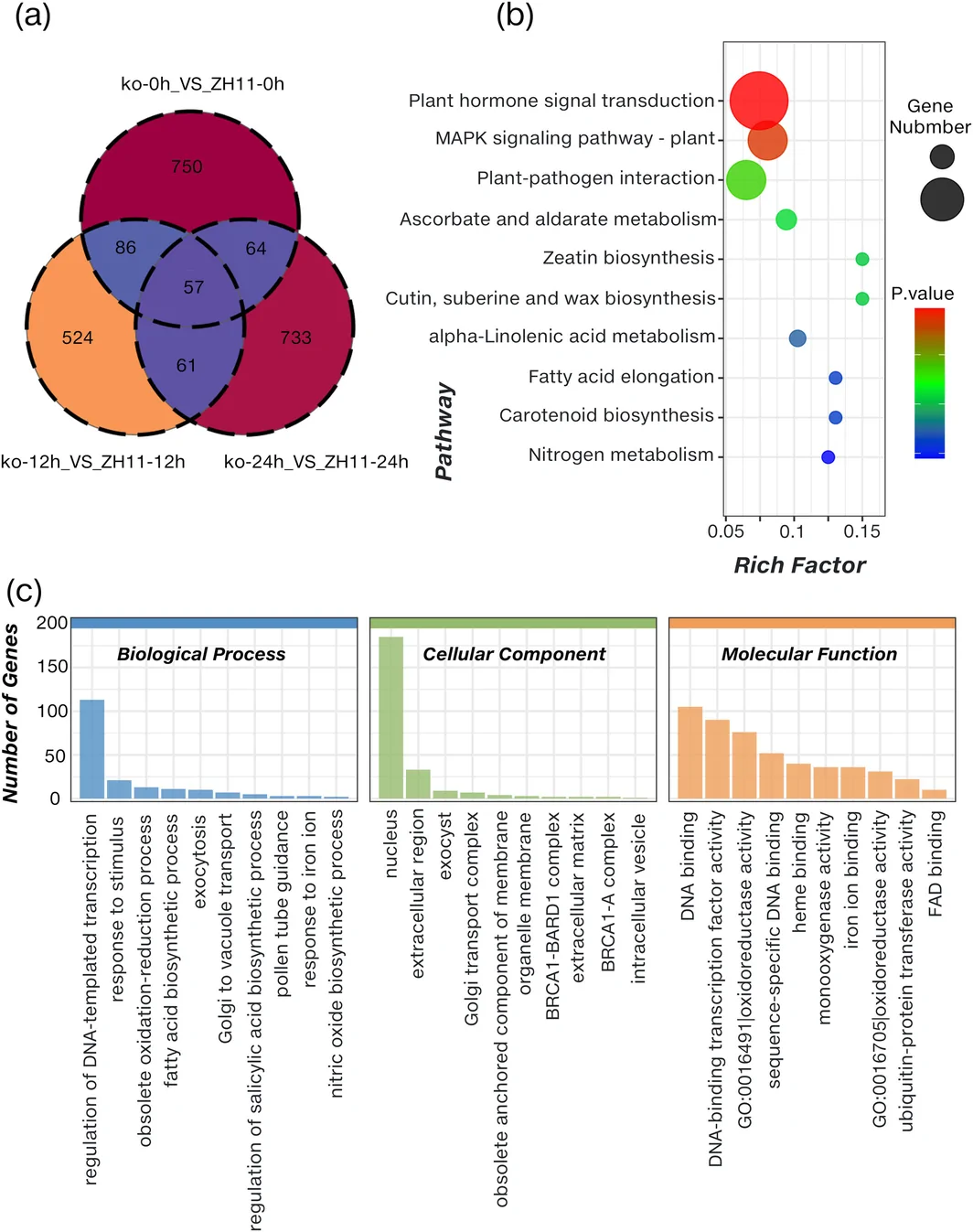
Transcriptomic analysis of *OsCBP609*‐dependent responses to *Magnaporthe oryzae* infection. (a) Venn diagram of differentially expressed genes (DEGs) between the *oscbp609* knockout mutant and Zhonghua 11 (ZH11) at 0, 12 and 24 h post‐inoculation (hpi). The 750 DEGs detected exclusively at 0 h were analysed separately as basal genotype‐dependent DEGs and were excluded from the DEG set used for the analyses in (b) and (c). (b) KEGG pathway enrichment analysis of the remaining 1525 DEGs. Bubble size indicates gene number, and colour represents the *p*‐value. (c) GO classification of the remaining 1525 DEGs into biological process, cellular component and molecular function categories.

Together, these transcriptomic analyses indicate that loss of *OsCBP609* is associated with altered transcriptional responses during *M. oryzae* infection, including changes in genes assigned to plant–pathogen interaction, MAPK signalling and hormone‐related pathways.

### 2.5 Loss of *OsCBP609* Impairs SA‐Responsive Defence Signalling

Because CBP60 proteins have been implicated in SA regulation in other plant species, we next examined whether the transcriptomic changes involving hormone‐related pathways were accompanied by altered SA homeostasis or responsiveness. Expression analysis of several SA biosynthesis‐related PAL genes revealed no coordinated transcriptional upregulation in the *oscbp609* mutant (Figure S5). We therefore next measured endogenous SA levels in four‐leaf‐stage plants under non‐inoculated conditions. Unexpectedly, basal SA levels were significantly higher in *ko‐oscbp609‐1* than in wild‐type ZH11 plants (Figure 5a), indicating that its enhanced susceptibility was not associated with reduced basal SA accumulation.

**FIGURE 5:**
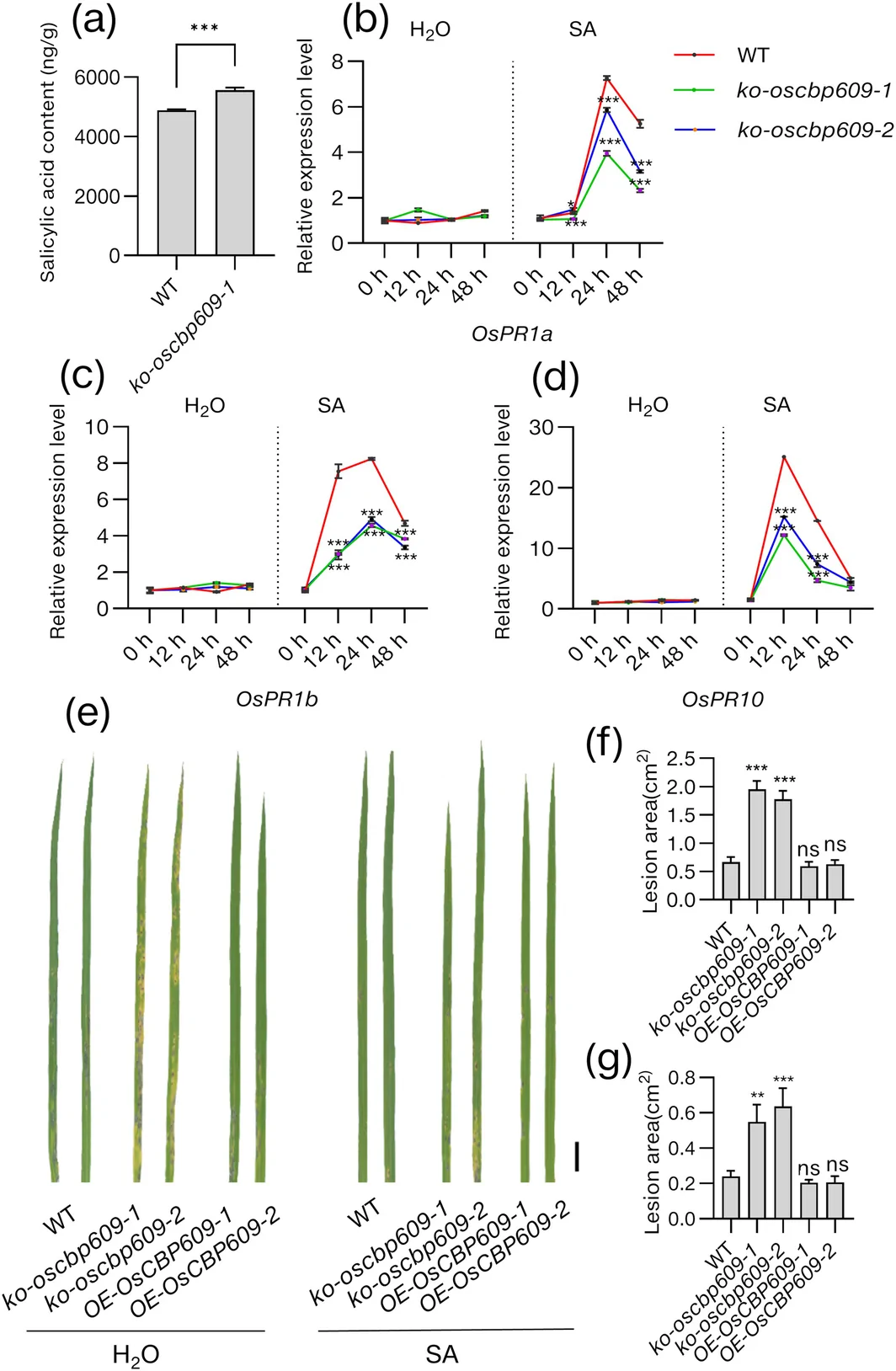
Loss of *OsCBP609* alters salicylic acid (SA)‐responsive defence in rice. (a) Endogenous SA content in wild‐type Zhonghua 11 (ZH11) and knockout mutant line *ko‐oscbp609‐1*. (b–d) Relative expression levels of *OsPR1a* (b), *OsPR1b* (c) and *OsPR10* (d) in ZH11 and two independent *oscbp609* knockout lines following water or SA treatment. Asterisks indicate significant differences compared with ZH11 at the corresponding time point and treatment. (e) Disease phenotypes of ZH11, two independent *oscbp609* knockout lines, and two independent *OsCBP609*‐overexpressing (OE) lines pretreated with water or SA before inoculation with *Magnaporthe oryzae* isolate ZJ21X18. (f, g) Quantification of lesion area following water (f) or SA (g) pretreatment. Data are presented as mean ± SD. **p < 0.01, ***p < 0.001; ns, not significant. Scale bar = 1 cm.

We next examined whether the mutants retained a normal transcriptional response to SA. WT and *oscbp609* seedlings were treated with 100 μM SA, and the expression of the SA‐responsive genes *OsPR1a*, *OsPR1b* and *OsPR10* was analysed at 0, 12, 24 and 48 h after treatment. Compared with ZH11, the induction of these genes was lower in the *oscbp609* mutants at one or more time points (Figure 5b–d). Thus, *OsCBP609* is not absolutely required for SA responsiveness but is required for the full induction of SA‐responsive defence genes.

To determine whether exogenous SA could compensate for the loss of *OsCBP609*, ZH11, the two *oscbp609* knockout lines and the two *OsCBP609*‐overexpressing lines were pretreated with SA or H_2_O before inoculation with *M. oryzae* isolate ZJ21X18. SA pretreatment reduced disease symptoms in all genotypes compared with their corresponding water‐treated controls, indicating that SA responsiveness was retained in the *oscbp609* mutants. However, the SA‐treated mutants remained significantly more susceptible than SA‐treated ZH11 plants (Figure 5e–g), indicating that exogenous SA could only partially compensate for the loss of *OsCBP609*. Together with the attenuated induction of *OsPR1a*, *OsPR1b* and *OsPR10*, these results indicate that OsCBP609 is required for the full transcriptional and disease‐resistance output of SA‐responsive immunity.

### 2.6 OsCBP609 Interacts With Pik1‐H4 in the Nucleus

To identify proteins potentially interacting with OsCBP609, we performed yeast two‐hybrid (Y2H) library screening. Among the candidate proteins recovered from the screen, Pik1‐H4 and four calmodulins (CaM1‐1, CaM1‐2, CaM2 and CaM3) were selected for pairwise testing because of their potential relevance to immune and Ca^2+^ signalling. Pairwise Y2H assays showed that OsCBP609 interacted with Pik1‐H4, CaM1‐1 and CaM1‐2, but not with CaM2 or CaM3 (Figure 6a). BD‐Pik1‐H4 exhibited autoactivation on SD/−Leu/−Trp/−His medium; however, this background growth was suppressed on stringent selective medium containing X‐α‐Gal and 200 ng mL^−1^ aureobasidin A (AbA). Under the same stringent conditions, yeast cells co‐expressing AD‐OsCBP609 and BD‐Pik1‐H4 grew and developed blue coloration, whereas the corresponding negative controls did not, supporting an interaction between OsCBP609 and Pik1‐H4. Consistently, firefly luciferase complementation imaging (LCI) produced strong luminescence in *N. benthamiana* leaves co‐expressing OsCBP609‐cLUC and Pik1‐H4‐nLUC, whereas no detectable signal was observed in the negative controls (Figure 6b).

**FIGURE 6:**
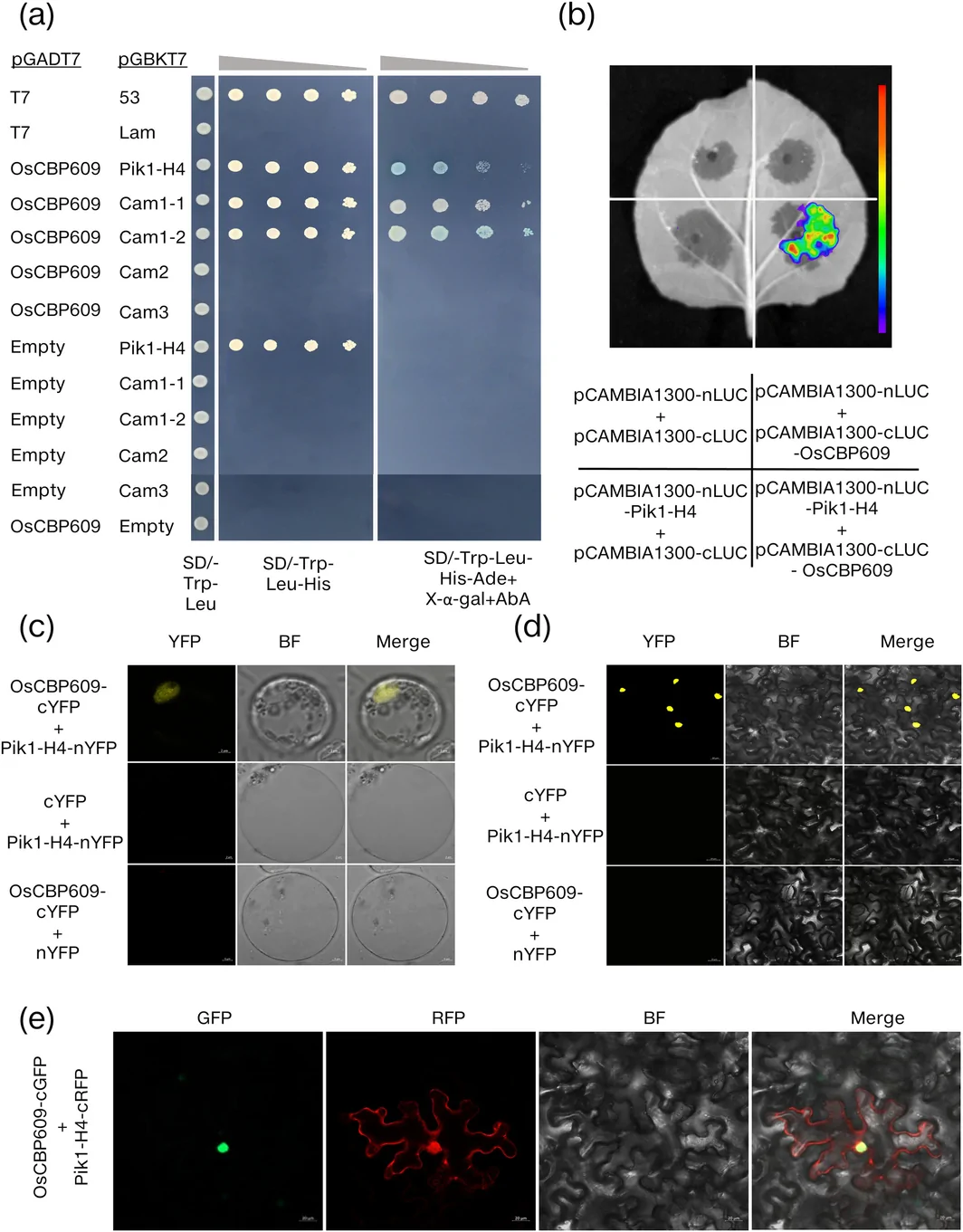
Interaction assays of OsCBP609 with Pik1‐H4 and selected calmodulins. (a) Yeast two‐hybrid (Y2H) assays showing interactions between OsCBP609 and Pik1‐H4, OsCBP609 and CaM1‐1, and OsCBP609 and CaM1‐2. (b) Luciferase complementation imaging (LCI) assays confirming the interaction between OsCBP609 and Pik1‐H4 in *Nicotiana benthamiana* leaves. The lower‐right quadrant represents the test section, with remaining sectors serving as controls. (c, d) Bimolecular fluorescence complementation (BiFC) assays detecting interactions between OsCBP609 and Pik1‐H4 in rice protoplasts (c) and *N. benthamiana* epidermal cells (d). (e) Subcellular co‐localization of OsCBP609 and Pik1‐H4 in *N. benthamiana* cells.

Bimolecular fluorescence complementation (BiFC) assays revealed clear YFP fluorescence in the nuclei of both *N. benthamiana* epidermal cells and rice protoplasts co‐expressing OsCBP609‐cYFP and Pik1‐H4‐nYFP (Figure 6c,d). Consistently, subcellular co‐localization analysis showed that OsCBP609‐cGFP and Pik1‐H4‐cRFP signals overlapped in the nucleus (Figure 6e). Together, these results demonstrate that OsCBP609 physically interacts with Pik1‐H4 in the nucleus.

We next examined whether co‐expression of OsCBP609 and Pik1‐H4 affected transcriptional activation. In the *N. benthamiana* LUC reporter system, Pik1‐H4 alone activated the mini 35S‐driven LUC reporter relative to the negative control (Figure S6a,b). Co‐expression of OsCBP609 and Pik1‐H4 produced significantly higher reporter activity than the expression of either protein alone (Figure S6d,e), indicating that co‐expression of the two proteins increases reporter activity.

### 2.7 Disruption of *OsCBP609* Reduces Blast Resistance in a *Pik*‐ *H4* Near‐Isogenic Line Background

Having established the physical interaction between OsCBP609 and Pik1‐H4, we next examined their functional relationship in a *Pik‐H4* near‐isogenic line (NIL) genetic background. We first assessed whether *Pik1‐H4* overexpression was associated with changes in *OsCBP609* expression using *Pik1‐H4*‐overexpressing plants generated in the LTH background. The transcript abundance of *OsCBP609* was significantly higher in the *Pik1‐H4*‐overexpressing lines than in wild‐type LTH plants (Figure S6c), indicating that *Pik1‐H4* overexpression is associated with increased *OsCBP609* expression.

To determine whether the *Pik‐H4* background affected *OsCBP609* expression in response to defence‐related signals, LTH and *Pik‐H4* NIL seedlings were treated with abscisic acid (ABA), 1‐aminocyclopropane‐1‐carboxylic acid (ACC), brassinosteroid (BR), jasmonic acid (JA), SA, or CaCl_2_. Following ABA, ACC, BR and JA treatments, *OsCBP609* exhibited genotype‐dependent temporal expression patterns, with the largest differences between LTH and the *Pik‐H4* NIL observed after JA and ACC treatment (Figure S7). Following SA treatment, *OsCBP609* expression showed pronounced temporal changes in the *Pik‐H4* NIL but remained relatively stable in LTH (Figure 7a). CaCl_2_ treatment also induced a transient increase in *OsCBP609* expression at 4 h in the *Pik‐H4* NIL, whereas no comparable increase was observed in LTH (Figure 7b). These results indicate that the expression pattern of *OsCBP609* in response to defence‐related signals differs between LTH and the *Pik‐H4* NIL.

**FIGURE 7:**
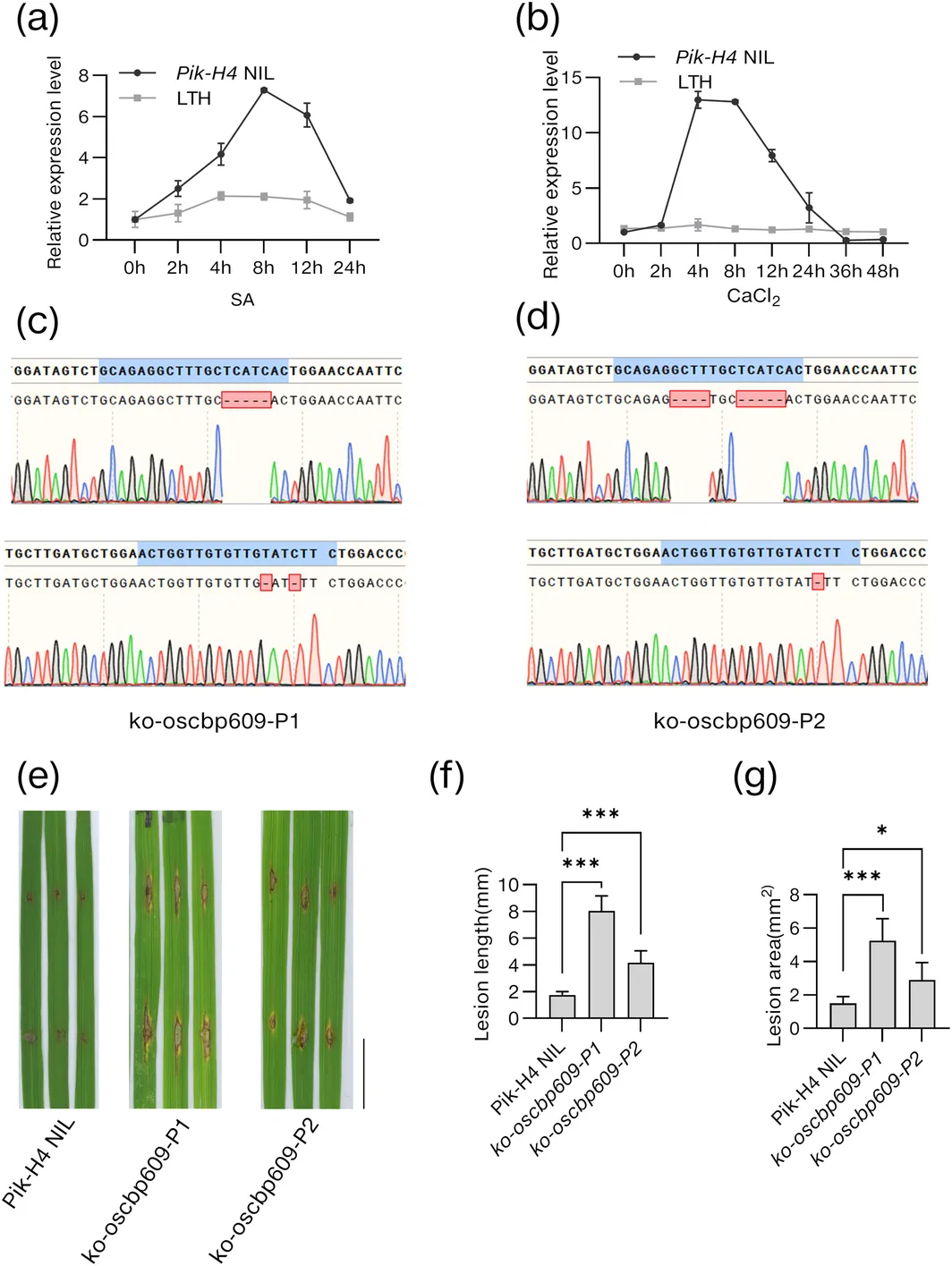
Disruption of *OsCBP609* reduces blast resistance in the *Pik‐H4* near‐isogenic line (NIL) background. (a, b) Relative expression of *OsCBP609* in Lijiangxintuanheigu (LTH) and the *Pik‐H4* NIL following salicylic acid (SA) (a) or CaCl_2_ (b) treatment. (c, d) Sanger sequencing chromatograms showing CRISPR/Cas9‐induced mutations in *OsCBP609* in two independent *Pik‐H4* NIL knockout lines, *ko‐oscbp609‐P1* (c) and *ko‐oscbp609‐P2* (d). Red boxes indicate the edited sites. (e) Disease phenotypes of the *Pik‐H4* NIL and knockout lines at 7 days post‐inoculation with *Magnaporthe oryzae* isolate ZJ21X18. (f, g) Lesion length (f) and lesion area (g). Data are presented as mean ± SD. *p < 0.05, ***p < 0.001. Scale bar = 1 cm.

To examine the contribution of *OsCBP609* to Pik‐H4‐associated resistance, *OsCBP609* was disrupted in the *Pik‐H4* NIL using a dual‐target CRISPR/Cas9 construct. Two independent homozygous edited lines, *ko‐oscbp609‐P1* and *ko‐oscbp609‐P2*, were selected for subsequent phenotypic analyses. Following inoculation with ZJ21X18, the independent knockout lines developed more severe disease symptoms, including significantly longer lesions and larger lesion areas than the unedited *Pik‐H4* NIL control (Figure 7c–g). These findings indicate that OsCBP609 contributes to blast resistance in the *Pik‐H4* genetic background and provide genetic support for the biological relevance of the OsCBP609–Pik1‐H4 association. Importantly, because disruption of OsCBP609 also compromises blast resistance in the ZH11 background, OsCBP609 is not restricted to Pik‐H4‐dependent resistance but appears to have a broader role in rice immunity.

## 3 Discussion

### 3.1 OsCBP609 Is a CBP60 Transcriptional Regulator Required for Effective Blast Resistance

CBP60 proteins are plant‐specific transcriptional regulators with important but functionally diverse roles in immunity. In *A. thaliana* , CBP60g and SARD1 positively regulate defence‐associated transcription and SA signalling, whereas other family members can have distinct or opposing functions (Wang et al. 2009, 2011; Zhang et al. 2010; Wan et al. 2012; Truman et al. 2013). More recent studies have identified the CBP60b clade as an evolutionarily conserved group of immune transcription factors whose activity depends on both DNA‐binding and calmodulin‐binding domains (Li et al. 2021, 2024). Our results extend the functional relevance of this family to rice by identifying *OsCBP609* as a nuclear‐localized transcriptional activator required for effective resistance to *M. oryzae* .

Disruption of *OsCBP609* increased lesion development and fungal biomass after inoculation with multiple *M. oryzae* isolates, whereas overexpression did not further enhance resistance relative to the WT. These results indicate that *OsCBP609* is required for normal immune competence but may not be rate‐limiting for blast resistance. Its activity may depend on interacting proteins, pathogen‐induced regulation, or functional redundancy with other CBP60 family members.

The calmodulin‐binding feature of CBP60 proteins also suggests a possible link to Ca^2+^‐dependent immune signalling. In our study, *OsCBP609* responded to CaCl_2_ treatment, Ca^2+^‐associated genes were altered in the mutant transcriptome, and CaM1‐1 and CaM1‐2 were identified as interacting proteins. These observations are consistent with a possible Ca^2+^/CaM‐related role, although direct regulation of *OsCBP609* by Ca^2+^/CaM remains to be demonstrated.

Neither knockout nor overexpression caused detectable changes in the agronomic traits examined, suggesting that altered *OsCBP609* expression did not cause major developmental defects under the conditions tested. However, broader evaluation of yield and field performance will be required before excluding possible fitness costs.

### 3.2 Loss of OsCBP609 Uncouples Basal SA Accumulation From SA‐Responsive Transcription

A major finding of this study is that basal SA accumulation and SA‐responsive transcription are uncoupled in the *oscbp609* mutants. Rice maintains relatively high basal SA levels, and both SA homeostasis and downstream SA signalling contribute to disease resistance (Silverman et al. 1995; Shimono et al. 2007; Liang et al. 2022; Xu et al. 2023). OsNPR1/NH1 is a central regulator of SA‐associated defence in rice (Chern et al. 2005; Yuan et al. 2007; Sugano et al. 2010). Consistent with this framework, reduced induction of *OsPR1a*, *OsPR1b* and *OsPR10* after SA treatment indicates that *oscbp609* plants have a diminished capacity to convert an SA stimulus into downstream defence transcription.

The elevated basal SA level in the *oscbp609* mutant argues against increased susceptibility being caused simply by insufficient SA accumulation. Instead, *OsCBP609* appears to contribute positively to SA‐responsive transcription while having a more complex role in SA homeostasis. The mechanism underlying the elevated basal SA level in the *oscbp609* mutant remains unresolved. Thus, the present data do not establish a direct role for OsCBP609 in SA biosynthesis or metabolism.

Exogenous SA reduced disease symptoms in all tested genotypes, showing that SA responsiveness was retained in the *oscbp609* mutants. However, SA‐treated mutants remained more susceptible than SA‐treated ZH11 plants and induction of *OsPR1a*, *OsPR1b* and *OsPR10* was attenuated. These findings indicate that *OsCBP609* is not absolutely required for SA signalling but is required for its full transcriptional and disease‐resistance output. Because the present experiments do not establish direct promoter binding, the examined PR genes should be regarded as downstream SA‐responsive markers rather than confirmed direct targets of *OsCBP609*.

### 3.3 OsCBP609 Is Functionally Associated With Pik‐H4‐Mediated Immunity

A second aspect of this study is the association between OsCBP609 and the NLR protein Pik1‐H4. Pik‐1 proteins mediate allele‐specific recognition of AVR‐Pik effectors through their integrated HMA domains (Ashikawa et al. 2008; Kanzaki et al. 2012; Maqbool et al. 2015; De la Concepcion et al. 2019). Our Y2H, LCI, BiFC and co‐localisation assays support a physical interaction between OsCBP609 and Pik1‐H4 in the nucleus. Co‐expression of the two proteins also produced greater reporter activity than expression of either protein alone, although the endogenous transcriptional targets of this complex remain unknown.

Disruption of *OsCBP609* in the *Pik‐H4* near‐isogenic background reduced blast resistance, providing genetic evidence that *OsCBP609* contributes to resistance in this genetic context. In addition, *OsCBP609* expression was elevated in *Pik1‐H4*‐overexpressing plants and showed distinct responses to defence‐related treatments in LTH and the *Pik‐H4* NIL. Together, these results support a functional association between OsCBP609 and Pik‐H4‐mediated immunity, consistent with the broader view that NLR‐mediated resistance depends on coordinated signalling and transcriptional regulation (Yuan, Jiang, et al. 2021; Ngou et al. 2021). *Pik‐H4* is a paired NLR locus with coordinated expression and function in rice blast resistance (Gu et al. 2025).

This association is also notable because CBP60 proteins have previously been linked to NLR‐dependent immune regulation, and fungal effectors can target CBP60‐family regulators (Li et al. 2021; Qin et al. 2018). However, the current data do not establish that OsCBP609 is a direct downstream component of Pik1‐H4 signalling. Importantly, the SA experiments were performed in the ZH11 background, which does not carry a functional *Pik‐H4* allele. Therefore, the SA‐responsive phenotype and the Pik1‐H4‐associated phenotype should be regarded as related but not yet linearly connected aspects of OsCBP609 function.

In summary, *OsCBP609* is a rice CBP60 transcriptional regulator required for effective resistance to *M. oryzae* . It supports SA‐responsive defence despite elevated basal SA accumulation in the mutant and is also physically and functionally associated with Pik1‐H4‐mediated immunity. Identifying the direct transcriptional targets of *OsCBP609* will be important for clarifying how these two aspects of its immune function are coordinated.

## 4 Experimental Procedures

### 4.1 Plant Materials and Growth Conditions

Rice varieties ZH11, LTH and the *Pik‐H4* NIL in the LTH background were used in this study. The *Pik‐H4* NIL carries the blast resistance locus *Pik‐H4*. ZH11 does not carry a functional *Pik‐H4* allele. To investigate the function of *OsCBP609*, two independent knockout lines, *ko‐oscbp609‐1* and *ko‐oscbp609‐2*, were generated in the ZH11 background using a single guide RNA targeting the coding region of *OsCBP609*. To examine the contribution of *OsCBP609* to Pik‐H4‐associated resistance, two guide RNAs targeting distinct sites within the *OsCBP609* coding region were assembled into a dual‐target CRISPR/Cas9 construct and introduced into the *Pik‐H4* NIL by *Agrobacterium tumefaciens* ‐mediated transformation. Regenerated plants were genotyped by PCR amplification and Sanger sequencing of the target regions. Two independent homozygous edited lines, *ko‐oscbp609‐P1* and *ko‐oscbp609‐P2*, were selected for subsequent analyses.

For overexpression, the full‐length coding sequence of *OsCBP609* (*LOC_Os02g08120*) was cloned into the pRHVcFlag vector, and the resulting construct was introduced into ZH11 by *A. tumefaciens* ‐mediated transformation to generate OE‐OsCBP609 plants. Primer sequences used for vector construction and genotyping are listed in Table S2.

Rice plants were grown in a greenhouse under a natural photoperiod at 30°C during the light period and 26°C during the dark period. *N. benthamiana* plants were used for transient expression assays. Seeds were germinated on Murashige and Skoog medium, and the resulting seedlings were transplanted into soil and maintained in a controlled growth chamber under a 16‐h light/8‐h dark photoperiod at 24°C.

### 4.2 Phylogenetic Analysis

Full‐length amino acid sequences were aligned using MAFFT (Katoh and Standley 2013). Maximum‐likelihood phylogenetic analysis was performed using IQ‐TREE v. 2 (Minh et al. 2020). The best‐fit amino acid substitution model was selected using ModelFinder (Kalyaanamoorthy et al. 2017) according to the Bayesian information criterion, which identified JTT + R4 as the optimal model. Branch support was assessed using 1000 ultrafast bootstrap replicates (Hoang et al. 2018).

### 4.3 Pathogen Inoculation and Disease Evaluation

The *M. oryzae* isolates Guy11, GDYJ7, Zhong1 and ZJ21X18 were used for inoculation experiments in this study. Conidial suspensions (5 × 10^5^ conidia/mL in 0.02% Tween 20) were spray‐inoculated onto 4‐leaf‐stage seedlings. Plants were incubated in a dew chamber at 26°C in the dark for 24 h, followed by 7 days under 26°C, 80% relative humidity, and a 12‐h light/12‐h dark cycle. Detached‐leaf punch inoculation was performed as described by Li et al. (2017). 5 μL of conidial suspension was applied to each wound site, and leaves were incubated in 10 μg/mL 6‐benzylaminopurine solution. Disease symptoms were recorded at 7 dpi. Lesion areas were quantified from digital images using ImageJ (Schneider et al. 2012), and fungal biomass was measured as described by Park et al. (2012).

### 4.4 RNA Extraction and RT‐qPCR Analysis

Total RNA was extracted from rice tissues using TRIzol reagent (Thermo) following the manufacturer's protocol, and cDNA was synthesized with the Evo M‐MLV RT Mix Kit (Accurate). qPCR was performed with AceQ SYBR Green Master Mix (Vazyme) using a Real‐Time PCR System (Applied Biosystems StepOnePlus). The β*‐actin* gene (*LOC_Os03g50885*) was used as an internal reference (Luo et al. 2023) and relative expression levels were calculated using the 2^−ΔΔ*C*t^ method (Livak and Schmittgen 2001).

### 4.5 Subcellular Localization

The full‐length CDS of *OsCBP609* was cloned into the pAN580 vector and transiently expressed in rice protoplasts together with a nuclear marker using the polyethylene glycol (PEG)‐mediated method (Zhang et al. 2011; Zhao et al. 2024). To test subcellular localization in planta, the CDSs of *OsCBP609* and *Pik1‐H4* were cloned into pEAQ‐cGFP and pEAQ‐cRFP vectors, respectively, generating OsCBP609‐cGFP and Pik1‐H4‐cRFP fusions. *A. tumefaciens* GV3101 strains harbouring the constructs were mixed (1:1, v/v) and co‐infiltrated into 4‐week‐old *N. benthamiana* leaves. Fluorescence signals were examined 36–48 h after infiltration with a confocal laser scanning microscope (Zhu et al. 2024).

### 4.6 Luciferase Reporter Assays

The CDSs of *OsCBP609* and *Pik1‐H4* were cloned into the pBD vector to generate effector constructs. pBD‐Empty, pBD‐OsCBP609 and pBD‐Pik1‐H4 driven by the CaMV 35S promoter served as effectors, while pGreenII 0800‐LUC‐GAL‐TATA carrying 5 × GAL4 motifs and mini 35S‐driven LUC served as the reporter (Zhu et al. 2024). Effector and reporter constructs were co‐infiltrated into *N. benthamiana* leaves via *A. tumefaciens* ‐mediated transient transformation. At 2–3 days after infiltration, the luciferase substrate was applied to the back of leaves, and luminescence signals were imaged using a NightSHADE LB985 plant imaging system (Berthold). Luminescence intensity was quantified from three independent biological replicates under identical imaging conditions.

### 4.7 Y2H and BiFC Assays

To assess transcriptional activation activity in yeast, pGBKT7‐Empty, pGBKT7‐IPA1 and pGBKT7‐OsCBP609 were individually transformed into Y2HGold cells. Transformants were grown on SD/−Trp, SD/−Trp/−His, SD/−Trp/−His/−Ade and SD/−Trp/−His/−Ade medium supplemented with X‐α‐Gal. For pairwise Y2H assays, the full‐length coding sequence of *OsCBP609* was cloned into pGADT7, whereas the full‐length coding sequences of *Pik1‐H4*, *CaM1‐1*, *CaM1‐2*, *CaM2* and *CaM3* were individually cloned into pGBKT7, generating AD‐OsCBP609 and the corresponding BD fusion constructs. The indicated plasmid combinations were co‐transformed into yeast cells according to the manufacturer's instructions (Clontech). Transformants were selected on SD/−Leu/−Trp medium and subsequently tested on SD/−Leu/−Trp/−His medium and SD/−Leu/−Trp/−His/−Ade medium supplemented with X‐α‐Gal and 200 ng mL^−1^ AbA.

For BiFC analysis, the CDSs of *OsCBP609* and *Pik1‐H4* were fused to cYFP and nYFP fragments in the pEAQ vector system to generate OsCBP609‐cYFP and Pik1‐H4‐nYFP, which were co‐expressed in *N. benthamiana* leaves (He et al. 2023). To confirm their interaction in rice cells, the CDSs were also introduced into pSAT6‐cYC and pSAT6‐cYN vectors and delivered into rice protoplasts by PEG‐mediated transformation. YFP fluorescence was examined using a confocal microscope.

### 4.8 Firefly LCI

The CDSs of *OsCBP609* and *Pik1‐H4* were cloned into pCAMBIA1300‐cLUC and pCAMBIA1300‐nLUC, respectively. The resulting constructs were transformed into *A. tumefaciens* GV3101, and equal volumes of bacterial suspensions were mixed and infiltrated into *N. benthamiana* leaves. Luminescence signals were detected using the same procedure as in the luciferase reporter assays (Wang et al. 2022).

### 4.9 Hormone and Ca^2+^ Treatments

For hormone‐ and Ca^2+^‐response assays, four‐leaf‐stage LTH and *Pik‐H4* NIL seedlings were sprayed with 100 μM SA, 100 μM JA, 1 mM ACC, 100 μM ABA, 100 μM BR, or 30 mM CaCl_2_. Leaves were harvested at the indicated time points, immediately frozen in liquid nitrogen, and stored at −80°C until RNA extraction for RT‐qPCR analysis. For SA signalling analysis, four‐leaf‐stage seedlings of wild‐type (ZH11) and *oscbp609* mutants were sprayed with 100 μM SA or double‐distilled water. Leaves were harvested at 0, 12, 24 and 48 h after treatment, immediately frozen in liquid nitrogen, and used for RT‐qPCR analysis of *OsPR1a*, *OsPR1b* and *OsPR10*. For disease resistance assays, four‐leaf‐stage ZH11, two *oscbp609* knockout lines and two *OsCBP609*‐overexpressing lines were treated with 100 μM SA or double‐distilled water as the control. After 24 h, the seedlings were spray‐inoculated with *M. oryzae* isolate ZJ21X18 as described above, and disease phenotypes were evaluated at 7 dpi.

### 4.10 Transcriptome Analysis

For RNA‐seq analysis, four‐leaf‐stage ZH11 and *oscbp609* seedlings were spray‐inoculated with *M. oryzae* isolate ZJ21X18, with 0.02% Tween 20 used as the mock treatment. Leaf samples were collected at 0, 12 and 24 hpi, with three independent biological replicates for each genotype and time point. RNA sequencing and subsequent bioinformatic analyses were performed by LC Bio Technologies Co. Ltd. (Hangzhou, China). DEGs were identified using DESeq2 (Love et al. 2014) on the company's bioinformatics platform, with thresholds of *p* < 0.05 and fold change > 2. Gene Ontology (GO) and Kyoto Encyclopedia of Genes and Genomes (KEGG) enrichment analyses were also performed using the company's bioinformatics platform.

### 4.11 Determination of Endogenous Salicylic Acid Content

Endogenous SA content was measured in leaves collected from four‐leaf‐stage ZH11 and *ko‐oscbp609‐1* seedlings under non‐inoculated conditions. SA was quantified using UPLC–MS/MS (PerkinElmer) with isotope‐labelled internal standards. Three independent biological replicates were analysed for each genotype.

### 4.12 Statistical Analysis

Statistical analyses were performed using GraphPad Prism v. 5. Data are presented as the mean ± SD from at least three independent biological replicates. Comparisons between two groups were performed using a two‐tailed Student's *t*‐test, whereas comparisons among multiple groups were conducted using one‐way ANOVA followed by Tukey's multiple‐comparison test.

## Author Contributions

**Huabin Xie:** conceptualization, methodology, visualization, writing – original draft, writing – review and editing, data curation, software, formal analysis. **Danhong Wei:** conceptualization, methodology, validation, investigation, data curation, visualization, writing – original draft. **Jiayang Li:** conceptualization, investigation, validation. **Chunzi Lin:** conceptualization, investigation, validation. **Xiaodi Zou:** investigation. **Renhui Li:** investigation, formal analysis. **Yan Hou:** writing – review and editing, visualization. **Xianya Jiang:** investigation, project administration, resources. **Chun Chen:** project administration, resources, supervision, conceptualization, methodology. **Tao Guo:** conceptualization, methodology, project administration, resources. **Jiafeng Wang:** conceptualization, methodology, funding acquisition, project administration, resources, supervision, writing – review and editing.

## Funding

This work was supported by the National Key Research and Development Program of China (2023YFD1400205‐1) China and the Innovation Fund of the Radiation‐Resistant Application Technology Innovation Center, China Institute of Atomic Energy (KFZC2025010401).

## Conflicts of Interest

The authors declare no conflicts of interest.

## Supporting information


**Figure S1:** The expression levels of defence‐related genes (*OsPR1a*, *OsPR1b* and *OsPAL1*) at 24 h post‐inoculation following inoculation with ZJ21X18. Data are presented as mean ± SD (*n* = 3). Significant differences are indicated as follows: *p < 0.05, **p < 0.01, ***p < 0.001; ns, not significant (Student's *t*‐test).

**Figure S2:** Altered *OsCBP609* expression has no detectable effect on the agronomic traits examined. (a) Comparison of grain shape among wild type (WT), *ko‐oscbp609* and OE‐OsCBP609. (b) Comparison of plant architecture among WT, *ko‐oscbp609* and OE‐OsCBP609. (c–f) Graphs of grain length (based on 10 grains), grain width (based on 10 grains), 1000‐grain weight and plant height for WT, *ko‐oscbp609* and *OE‐OsCBP609*, respectively. No significant differences were observed in these four traits among the three lines.

**Figure S3:** Reverse transcription‐quantitative PCR (RT‐qPCR) validation of eight randomly selected differentially expressed genes (DEGs) from RNA‐seq. (a–h) Time‐course expression analysis of eight selected DEGs in wild type (WT) and *ko‐oscbp609* plants at 0, 12 and 24 h post‐inoculation (hpi) with *Magnaporthe oryzae* , as validated by RT‐qPCR. Data represent mean ± SD (*n* = 3). Significant differences are indicated as follows: *p < 0.05, **p < 0.01, ***p < 0.001; ns, not significant (Student's *t*‐test).

**Figure S4:** KEGG enrichment analysis of the 750 basal genotype‐dependent differentially expressed genes (DEGs) detected exclusively in the ko‐0h_VS_ZH11‐0 h comparison.

**Figure S5:** Expression analysis of salicylic acid (SA) biosynthesis‐related PAL genes in ZH11, *oscbp609* knockout lines and *OsCBP609*‐overexpressing lines. Relative transcript levels of *OsPAL1*, *OsPAL2*, *OsPAL3* and *OsPAL6* were determined by reverse transcription‐quantitative PCR. Data represent mean ± SD (*n* = 3).

**Figure S6:** Co‐expression of *OsCBP609* and *Pik1‐H4* increases reporter activity. (a, b) Luciferase reporter assay showing the effect of Pik1‐H4 on reporter activity in *Nicotiana benthamiana* leaves, with quantitative analysis shown in (b). (c) Relative expression of *OsCBP609* in Lijiangxintuanheigu (LTH) and *Pik1‐H4*‐overexpressing plants. (d, e) Luciferase reporter assay showing the effects of *OsCBP609* and *Pik1‐H4* co‐expression on reporter activity, with quantitative analysis shown in (e).

**Figure S7:** Dynamic transcriptional response of *OsCBP609* to exogenous hormone treatments. (a–d) Transcript levels of *OsCBP609* in Lijiangxintuanheigu (LTH) and *Pik‐H4* NIL plants following treatment with jasmonic acid (JA, 100 μM), abscisic acid (ABA, 100 μM), 1‐aminocyclopropane‐1‐carboxylic acid (ACC, 1 mM) and brassinosteroid (BR, 100 μM), as determined by reverse transcription‐quantitative PCR. Data represent mean ± SD (*n* = 3).

**Table S1:** Inventory of differentially expressed genes (DEGs) in wild type (WT) and *ko‐cbp609* plants at 0 h (sheet 1), 12 h (sheet 2) and 24 h (sheet 3) post‐inoculation with ZJ21X18.

**Table S2:** Primers used in this study.

## Data Availability

The data that support the findings of this study are openly available in Genome Sequence Archive (GSA) at https://ngdc.cncb.ac.cn/gsa, reference number CRA032456.

## References

[mpp70384-bib-0001] Ashikawa, I. , N. Hayashi , H. Yamane , et al. 2008. “Two Adjacent Nucleotide‐Binding Site‐Leucine‐Rich Repeat Class Genes Are Required to Confer *Pikm*‐Specific Rice Blast Resistance.” Genetics 180: 2267–2276. 10.1534/genetics.108.095034. PMC2600957 18940787

[mpp70384-bib-0002] Azizi, P. , M. Y. Rafii , S. N. A. Abdullah , et al. 2016. “Toward Understanding of Rice Innate Immunity Against *Magnaporthe oryzae* .” Critical Reviews in Biotechnology 36: 165–174. 10.3109/07388551.2014.946883. 25198435

[mpp70384-bib-0003] Boudsocq, M. , M. R. Willmann , M. McCormack , et al. 2010. “Differential Innate Immune Signalling via Ca^2+^ Sensor Protein Kinases.” Nature 464: 418–422. 10.1038/nature08794. PMC2841715 20164835

[mpp70384-bib-0004] Chern, M. , H. A. Fitzgerald , P. E. Canlas , D. A. Navarre , and P. C. Ronald . 2005. “Overexpression of a Rice NPR1 Homolog Leads to Constitutive Activation of Defence Response and Hypersensitivity to Light.” Molecular Plant–Microbe Interactions 18: 511–520. 10.1094/mpmi-18-0511. 15986920

[mpp70384-bib-0005] De la Concepcion, J. C. , M. Franceschetti , D. MacLean , R. Terauchi , S. Kamoun , and M. J. Banfield . 2019. “Protein Engineering Expands the Effector Recognition Profile of a Rice NLR Immune Receptor.” eLife 8: e47713. 10.7554/eLife.47713. PMC6768660 31535976

[mpp70384-bib-0006] DeFalco, T. A. , K. W. Bender , and W. A. Snedden . 2010. “Breaking the Code: Ca^2+^ Sensors in Plant Signalling.” Biochemical Journal 425: 27–40. 10.1042/BJ20091147. 20001960

[mpp70384-bib-0007] Dodds, P. N. , and J. P. Rathjen . 2010. “Plant Immunity: Towards an Integrated View of Plant–Pathogen Interactions.” Nature Reviews Genetics 11: 539–548. 10.1038/nrg2812. 20585331

[mpp70384-bib-0008] Du, L. , G. S. Ali , K. A. Simons , et al. 2009. “Ca^2+^/Calmodulin Regulates Salicylic‐Acid‐Mediated Plant Immunity.” Nature 457: 1154–1158. 10.1038/nature07612. 19122675

[mpp70384-bib-0009] Elert, E. 2014. “Rice by the Numbers: A Good Grain.” Nature 514: S50–S51. 10.1038/514S50a. 25368886

[mpp70384-bib-0010] Gu, F. , H. Xie , Q. Huang , et al. 2025. “Co‐Expression Pattern Analysis of Head‐To‐Head NLR Gene Pair *Pik‐H4* .” Plant, Cell & Environment 48: 5342–5356. 10.1111/pce.15509. 40177737

[mpp70384-bib-0011] He, Y. , S. Sun , J. Zhao , et al. 2023. “UDP‐Glucosyltransferase OsUGT75A Promotes Submergence Tolerance During Rice Seed Germination.” Nature Communications 14: 2296. 10.1038/s41467-023-38085-5. PMC10121563 37085517

[mpp70384-bib-0012] Hoang, D. T. , O. Chernomor , A. von Haeseler , B. Q. Minh , and L. S. Vinh . 2018. “UFBoot2: Improving the Ultrafast Bootstrap Approximation.” Molecular Biology and Evolution 35: 518–522. 10.1093/molbev/msx281. PMC5850222 29077904

[mpp70384-bib-0013] Hu, G. , M. Hao , L. Wang , et al. 2020. “The Cotton miR477‐CBP60A Module Participates in Plant Defence Against *Verticillium dahliae* .” Molecular Plant–Microbe Interactions 33: 624–636. 10.1094/MPMI-10-19-0302-R 31868566

[mpp70384-bib-0014] Jones, J. D. G. , and J. L. Dangl . 2006. “The Plant Immune System.” Nature 444: 323–329. 10.1038/nature05286. 17108957

[mpp70384-bib-0015] Kalyaanamoorthy, S. , B. Q. Minh , T. K. F. Wong , A. von Haeseler , and L. S. Jermiin . 2017. “ModelFinder: Fast Model Selection for Accurate Phylogenetic Estimates.” Nature Methods 14: 587–589. 10.1038/nmeth.4285. PMC5453245 28481363

[mpp70384-bib-0016] Kanzaki, H. , K. Yoshida , H. Saitoh , et al. 2012. “Arms Race co‐Evolution of *Magnaporthe oryzae* *AVR‐Pik* and Rice *Pik* Genes Driven by Their Physical Interactions.” Plant Journal 72: 894–907. 10.1111/j.1365-313x.2012.05110.x. 22805093

[mpp70384-bib-0017] Katoh, K. , and D. M. Standley . 2013. “MAFFT Multiple Sequence Alignment Software Version 7: Improvements in Performance and Usability.” Molecular Biology and Evolution 30: 772–780. 10.1093/molbev/mst010. PMC3603318 23329690

[mpp70384-bib-0018] Li, L. , Y. Yang , Y. Chen , et al. 2024. “CBP60b Clade Proteins Are Prototypical Transcription Factors Mediating Immunity.” Plant Physiology 196: 1489–1501. 10.1093/plphys/kiae349. 38889048

[mpp70384-bib-0019] Li, L. , J. Ying , E. Li , et al. 2021. “ *Arabidopsis* CBP60b Is a Central Transcriptional Activator of Immunity.” Plant Physiology 186: 1645–1659. 10.1093/plphys/kiab164. PMC8260125 33848345

[mpp70384-bib-0020] Li, W. , Z. Zhu , M. Chern , et al. 2017. “A Natural Allele of a Transcription Factor in Rice Confers Broad‐Spectrum Blast Resistance.” Cell 170: 114–126.e15. 10.1016/j.cell.2017.06.008. 28666113

[mpp70384-bib-0021] Liang, B. , H. Wang , C. Yang , et al. 2022. “Salicylic Acid Is Required for Broad‐Spectrum Disease Resistance in Rice.” International Journal of Molecular Sciences 23: 1354. 10.3390/ijms23031354. PMC8836234 35163275

[mpp70384-bib-0022] Livak, K. J. , and T. D. Schmittgen . 2001. “Analysis of Relative Gene Expression Data Using Real‐Time Quantitative PCR and the 2^−ΔΔCT^ Method.” Methods 25: 402–408. 10.1006/meth.2001.1262. 11846609

[mpp70384-bib-0023] Love, M. I. , W. Huber , and S. Anders . 2014. “Moderated Estimation of Fold Change and Dispersion for RNA‐Seq Data With DESeq2.” Genome Biology 15: 550. 10.1186/s13059-014-0550-8. PMC4302049 25516281

[mpp70384-bib-0024] Luo, L. , Y. Xie , S. Yu , et al. 2023. “The DnaJ Domain‐Containing Heat‐Shock Protein NAL11 Determines Plant Architecture by Mediating Gibberellin Homeostasis in Rice ( *Oryza sativa* ).” New Phytologist 237: 2163–2179. 10.1111/nph.18696. 36564987

[mpp70384-bib-0025] Maqbool, A. , H. Saitoh , M. Franceschetti , et al. 2015. “Structural Basis of Pathogen Recognition by an Integrated HMA Domain in a Plant NLR Immune Receptor.” eLife 4: e08709. 10.7554/eLife.08709. PMC4547098 26304198

[mpp70384-bib-0026] Minh, B. Q. , H. A. Schmidt , O. Chernomor , et al. 2020. “IQ‐TREE 2: New Models and Efficient Methods for Phylogenetic Inference in the Genomic Era.” Molecular Biology and Evolution 37: 1530–1534. 10.1093/molbev/msaa015. PMC7182206 32011700

[mpp70384-bib-0027] Ngou, B. P. M. , H.‐K. Ahn , P. Ding , and J. D. G. Jones . 2021. “Mutual Potentiation of Plant Immunity by Cell‐Surface and Intracellular Receptors.” Nature 592: 110–115. 10.1038/s41586-021-03315-7. 33692545

[mpp70384-bib-0028] Park, C. H. , S. Chen , G. Shirsekar , et al. 2012. “The *Magnaporthe oryzae* Effector AvrPiz‐t Targets the RING E3 Ubiquitin Ligase APIP6 to Suppress Pathogen‐Associated Molecular Pattern‐Triggered Immunity in Rice.” Plant Cell 24: 4748–4762. 10.1105/tpc.112.105429. PMC3531864 23204406

[mpp70384-bib-0029] Qin, J. , K. Wang , L. Sun , et al. 2018. “The Plant‐Specific Transcription Factors CBP60g and SARD1 Are Targeted by a *Verticillium* Secretory Protein VdSCP41 to Modulate Immunity.” eLife 7: e34902. 10.7554/eLife.34902. PMC5993538 29757140

[mpp70384-bib-0030] Schneider, C. A. , W. S. Rasband , and K. W. Eliceiri . 2012. “NIH Image to ImageJ: 25 Years of Image Analysis.” Nature Methods 9: 671–675. 10.1038/nmeth.2089. PMC5554542 22930834

[mpp70384-bib-0031] Shimono, M. , S. Sugano , A. Nakayama , et al. 2007. “Rice WRKY45 Plays a Crucial Role in Benzothiadiazole‐Inducible Blast Resistance.” Plant Cell 19: 2064–2076. 10.1105/tpc.106.046250. PMC1955718 17601827

[mpp70384-bib-0032] Silverman, P. , M. Seskar , D. Kanter , P. Schweizer , J.‐P. Métraux , and I. Raskin . 1995. “Salicylic Acid in Rice (Biosynthesis, Conjugation, and Possible Role).” Plant Physiology 108: 633–639. 10.1104/pp.108.2.633. PMC157383 12228500

[mpp70384-bib-0033] Sugano, S. , C.‐J. Jiang , S.‐I. Miyazawa , et al. 2010. “Role of OsNPR1 in Rice Defence Program as Revealed by Genome‐Wide Expression Analysis.” Plant Molecular Biology 74: 549–562. 10.1007/s11103-010-9695-3 20924648

[mpp70384-bib-0034] Sun, T. , Y. Zhang , Y. Li , Q. Zhang , Y. Ding , and Y. Zhang . 2015. “ChIP‐Seq Reveals Broad Roles of SARD1 and CBP60g in Regulating Plant Immunity.” Nature Communications 6: 10159. 10.1038/ncomms10159. PMC4703862 27206545

[mpp70384-bib-0035] Truman, W. , S. Sreekanta , Y. Lu , et al. 2013. “The CALMODULIN‐BINDING PROTEIN60 Family Includes Both Negative and Positive Regulators of Plant Immunity.” Plant Physiology 163: 1741–1751. 10.1104/pp.113.227108. PMC3850189 24134885

[mpp70384-bib-0036] Wan, D. , R. Li , B. Zou , et al. 2012. “Calmodulin‐Binding Protein CBP60g Is a Positive Regulator of Both Disease Resistance and Drought Tolerance in *Arabidopsis* .” Plant Cell Reports 31: 1269–1281. 10.1007/s00299-012-1247-7. 22466450

[mpp70384-bib-0037] Wang, L. , K. Tsuda , M. Sato , J. D. Cohen , F. Katagiri , and J. Glazebrook . 2009. “ *Arabidopsis* CaM Binding Protein CBP60g Contributes to MAMP‐Induced SA Accumulation and Is Involved in Disease Resistance Against *Pseudomonas syringae* .” PLoS Pathogens 5: e1000301. 10.1371/journal.ppat.1000301. PMC2633612 19214217

[mpp70384-bib-0038] Wang, L. , K. Tsuda , W. Truman , et al. 2011. “CBP60g and SARD1 Play Partially Redundant Critical Roles in Salicylic Acid Signaling.” Plant Journal 67: 1029–1041. 10.1111/j.1365-313X.2011.04655.x. 21615571

[mpp70384-bib-0039] Wang, M. , X. Zhu , G. Peng , et al. 2022. “Methylesterification of Cell‐Wall Pectin Controls the Diurnal Flower‐Opening Times in Rice.” Molecular Plant 15: 956–972. 10.1016/j.molp.2022.04.004. 35418344

[mpp70384-bib-0040] Wang, Y. , Y. Lv , Y. Wen , et al. 2025. “GS2 Cooperates With IPA1 to Control Panicle Architecture.” New Phytologist 245: 2726–2743. 10.1111/nph.20412. PMC11840411 39887382

[mpp70384-bib-0041] Xiao, W. , Q. Yang , H. Wang , et al. 2011. “Identification and Fine Mapping of a Resistance Gene to *Magnaporthe oryzae* in a Space‐Induced Rice Mutant.” Molecular Breeding 28: 303–312. 10.1007/s11032-010-9481-6.

[mpp70384-bib-0042] Xu, L. , H. Zhao , J. Wang , et al. 2023. “AIM1‐Dependent High Basal Salicylic Acid Accumulation Modulates Stomatal Aperture in Rice.” New Phytologist 238: 1420–1430. 10.1111/nph.18842. 36843251

[mpp70384-bib-0043] Ye, M. , H. Lan , and J. Liu . 2024. “GmCBP60b Plays Both Positive and Negative Roles in Plant Immunity.” International Journal of Molecular Sciences 25: 378. 10.3390/ijms25010378. PMC10778643 38203547

[mpp70384-bib-0044] Yuan, M. , Z. Jiang , G. Bi , et al. 2021. “Pattern‐Recognition Receptors Are Required for NLR‐Mediated Plant Immunity.” Nature 592: 105–109. 10.1038/s41586-021-03316-6. PMC8016741 33692546

[mpp70384-bib-0045] Yuan, M. , B. P. M. Ngou , P. Ding , and X.‐F. Xin . 2021. “PTI–ETI Crosstalk: An Integrative View of Plant Immunity.” Current Opinion in Plant Biology 62: 102030. 10.1016/j.pbi.2021.102030. 33684883

[mpp70384-bib-0046] Yuan, Y. , S. Zhong , Q. Li , et al. 2007. “Functional Analysis of Rice NPR1‐Like Genes Reveals That OsNPR1/NH1 Is the Rice Orthologue Conferring Disease Resistance With Enhanced Herbivore Susceptibility.” Plant Biotechnology Journal 5: 313–324. 10.1111/j.1467-7652.2007.00243.x. 17309686

[mpp70384-bib-0047] Zeng, H. , L. Xu , A. Singh , H. Wang , L. Du , and B. W. Poovaiah . 2015. “Involvement of Calmodulin and Calmodulin‐Like Proteins in Plant Responses to Abiotic Stresses.” Frontiers in Plant Science 6: 600. 10.3389/fpls.2015.00600. PMC4532166 26322054

[mpp70384-bib-0048] Zhang, Y. , J. Su , S. Duan , et al. 2011. “A Highly Efficient Rice Green Tissue Protoplast System for Transient Gene Expression and Studying Light/Chloroplast‐Related Processes.” Plant Methods 7: 30. 10.1186/1746-4811-7-30. PMC3203094 21961694

[mpp70384-bib-0049] Zhang, Y. , S. Xu , P. Ding , et al. 2010. “Control of Salicylic Acid Synthesis and Systemic Acquired Resistance by Two Members of a Plant‐Specific Family of Transcription Factors.” Proceedings of the National Academy of Sciences of the United States of America 107: 18220–18225. 10.1073/pnas.1005225107. PMC2964219 20921422

[mpp70384-bib-0050] Zhao, J. , J. Shao , Z. Zeng , et al. 2024. “Knocking Out Isopropylmalate Synthase Simultaneously Improves Grain Appearance and Nutritional Quality in Rice.” Plant Journal 120: 159–173. 10.1111/tpj.16977. 39145531

[mpp70384-bib-0051] Zhu, X. , M. Wang , Z. Huang , et al. 2024. “The OsMYC2–JA Feedback Loop Regulates Diurnal Flower‐Opening Time via Cell Wall Loosening in Rice.” Plant Journal 119: 2585–2598. 10.1111/tpj.16910. 38972041

