# Supplementary material for: The Rice CBP60 Protein OsCBP609 Interacts With Pik1‐H4 and Supports Salicylic Acid‐Responsive Immunity Against Magnaporthe oryzae
Source: Mol Plant Pathol. 2026 Nov 8;27(11):e70384. doi: 10.1111/mpp.70384 (PMC13745637; doi:10.1111/mpp.70384)
Supplement: Supplementary file 1 [file MPP-27-e70384-s003.docx]

**Figure S5**

Expression analysis of SA biosynthesis-related PAL genes in ZH11, *oscbp609* knockout lines, and *OsCBP609*-overexpressing lines. Relative transcript levels of *OsPAL1*, *OsPAL2*, *OsPAL3*, and *OsPAL6* were determined by qRT-PCR. Data represent mean ± SD (n = 3).
