# Supplementary material for: The Rice CBP60 Protein OsCBP609 Interacts With Pik1‐H4 and Supports Salicylic Acid‐Responsive Immunity Against Magnaporthe oryzae
Source: Mol Plant Pathol. 2026 Nov 8;27(11):e70384. doi: 10.1111/mpp.70384 (PMC13745637; doi:10.1111/mpp.70384)
Supplement: Supplementary file 2 [file MPP-27-e70384-s004.docx]

**Figure S6**

Co-expression of OsCBP609 and Pik1-H4 increases reporter activity. (a, b) Luciferase reporter assay showing the effect of Pik1-H4 on reporter activity in N. benthamiana leaves, with quantitative analysis shown in (b). (c) Relative expression of *OsCBP609* in LTH and Pik1-H4-overexpressing plants. (d, e) Luciferase reporter assay showing the effects of OsCBP609 and Pik1-H4 co-expression on reporter activity, with quantitative analysis shown in (e).
