# Supplementary material for: The Rice CBP60 Protein OsCBP609 Interacts With Pik1‐H4 and Supports Salicylic Acid‐Responsive Immunity Against Magnaporthe oryzae
Source: Mol Plant Pathol. 2026 Nov 8;27(11):e70384. doi: 10.1111/mpp.70384 (PMC13745637; doi:10.1111/mpp.70384)
Supplement: Supplementary file 3 [file MPP-27-e70384-s005.docx]

**Figure S7**

Dynamic transcriptional response of *OsCBP609* to exogenous hormone treatments. (a–d) Transcript levels of *OsCBP609* in LTH and *Pik-H4* NIL plants following treatment with JA (100 μM), ABA (100 μM), ACC (1 mM) and BR (100 μM), as determined by qRT-PCR. Data represent mean ± SD (n = 3).
