# Supplementary material for: The Rice CBP60 Protein OsCBP609 Interacts With Pik1‐H4 and Supports Salicylic Acid‐Responsive Immunity Against Magnaporthe oryzae
Source: Mol Plant Pathol. 2026 Nov 8;27(11):e70384. doi: 10.1111/mpp.70384 (PMC13745637; doi:10.1111/mpp.70384)
Supplement: Supplementary file 4 [file MPP-27-e70384-s006.docx]

**Figure S3**

qRT-PCR validation of eight randomly selected DEGs from RNA-seq. (a–h) Time-course expression analysis of eight selected DEGs in WT and *ko-oscbp609* plants at 0, 12, and 24 hpi with *M. oryzae*, as validated by qRT-PCR. Data represent mean ± SD (n = 3). Significant differences are indicated as follows: **P* < 0.05, ***P* < 0.01, ****P* < 0.001; ns, not significant (Student's t-test).
