# Supplementary material for: The Rice CBP60 Protein OsCBP609 Interacts With Pik1‐H4 and Supports Salicylic Acid‐Responsive Immunity Against Magnaporthe oryzae
Source: Mol Plant Pathol. 2026 Nov 8;27(11):e70384. doi: 10.1111/mpp.70384 (PMC13745637; doi:10.1111/mpp.70384)
Supplement: Supplementary file 5 [file MPP-27-e70384-s007.docx]

**Figure S2**

Altered *OsCBP609* expression has no detectable effect on the agronomic traits examined. (a) Comparison of grain shape among WT, *ko-oscbp609*, and OE-OsCBP609. (b) Comparison of plant architecture among WT, *ko-oscbp609*, and OE-OsCBP609. (c-f) Statistical graphs of grain length (based on 10 grains), grain width (based on 10 grains), thousand-grain weight, and plant height for WT, *ko-oscbp609*, and *OE-OsCBP609*, respectively. No significant differences were observed in these four traits among the three lines.
