# Supplementary material for: The Rice CBP60 Protein OsCBP609 Interacts With Pik1‐H4 and Supports Salicylic Acid‐Responsive Immunity Against Magnaporthe oryzae
Source: Mol Plant Pathol. 2026 Nov 8;27(11):e70384. doi: 10.1111/mpp.70384 (PMC13745637; doi:10.1111/mpp.70384)
Supplement: Supplementary file 6 [file MPP-27-e70384-s008.docx]

**Figure S4**

KEGG enrichment analysis of the 750 basal genotype-dependent DEGs detected exclusively in the ko-0h_VS_ZH11-0h comparison.
