# Supplementary material for: The Rice CBP60 Protein OsCBP609 Interacts With Pik1‐H4 and Supports Salicylic Acid‐Responsive Immunity Against Magnaporthe oryzae
Source: Mol Plant Pathol. 2026 Nov 8;27(11):e70384. doi: 10.1111/mpp.70384 (PMC13745637; doi:10.1111/mpp.70384)
Supplement: Supplementary file 7 [file MPP-27-e70384-s009.docx]

**Figure S1**

The expression levels of defence-related genes (*OsPR1a*, *OsPR1b*, and *OsPAL1*) at 24 hpi following inoculation with ZJ21X18. Data are presented as mean ± SD (n = 3). Significant differences are indicated as follows: **P* < 0.05, ***P* < 0.01, ****P* < 0.001; ns, not significant (Student’s t-test).
